# Flaxseed Ingestion Alters Ratio of Enterolactone Enantiomers in Human Serum

**DOI:** 10.1155/2010/403076

**Published:** 2010-05-05

**Authors:** Niina M. Saarinen, Annika I. Smeds, José L. Peñalvo, Tarja Nurmi, Herman Adlercreutz, Sari Mäkelä

**Affiliations:** ^1^Functional Foods Forum, University of Turku, Itäinen Pitkäkatu 4A, 20014, Finland; ^2^Laboratory of Organic Chemistry, Åbo Akademi University, Biskopsgatan 8, 20500 Turku, Finland; ^3^Institute for Preventive Medicine, Nutrition and Cancer, Folkhälsan Research Center, and Division of Clinical Chemistry, University of Helsinki, PB 63, 00014, Helsinki, Finland; ^4^Research Institute of Public Health, School of Public Health and Clinical Nutrition, University of Kuopio, P.O. Box 1627, 70211 Kuopio, Finland; ^5^Institute of Biomedicine, University of Turku, 20014 Turku, Finland

## Abstract

Enterolactone (EL) is an enterolignan found in human subjects. In this pilot study, the enantiomeric ratios of serum EL were determined in serum from healthy adults during consumption of habitual diet, and after an 8-day supplementation with flaxseed (25 g/day). (−)EL dominated in all serum samples collected during habitual diet consumption. However, the ratio of (−)EL and (+)EL enantiomers differed markedly between individuals. Flaxseed ingestion increased significantly the proportion of (+)EL in all subjects. Moreover, a small but significant increase in serum (−)EL concentration was measured. After flaxseed ingestion, (−)EL concentrations correlated with those of (+)EL suggesting that the stereochemistry of the parent plant lignan in flaxseed is not a major determinant of EL formation in human subjects. Comparison of EL concentrations obtained with the validated chromatographic methods (HPLC-MS/MS, HPLC-CEAD, and GC-MS) and the time-resolved fluoroimmunoassay (TR-FIA) revealed that the immunoassay method underestimates human serum EL concentrations after the flaxseed ingestion.

## 1. Introduction

Enterolactone (EL) is an enterolignan produced by intestinal microbiota from several dietary plant lignans [1]. In several epidemiological studies, low serum and urine EL concentrations have been associated with an increased risk for breast, prostate, and colon cancers, and cardiovascular disease [2–6] suggesting a possible role of EL as a protective agent against the development of chronic diseases. 

**Figure 1:**
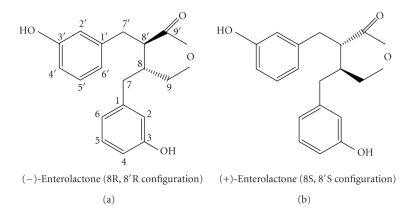
Chemical structure of enterolactone enantiomers.

 EL is a chiral compound, and the enantiomers occurring as plant lignan metabolites are (8R, 8′R)-(−)EL and (8S, 8′S)-(+)EL (Figure 1). Our previous studies in rats have demonstrated that the absolute configuration at C8 and C8′ of the parent plant lignan is not changed during the intestinal metabolism to EL [7]. Therefore, the ratio of (+) and (−) enantiomers of formed EL is determined by the stereochemistry of the plant lignan precursors. Whether the stereochemistry determines metabolic conversion capability of plant lignans to enterolignans is not yet known. 

 Since the first published report on the identification and quantification of EL in human plasma [8], several chromatographic methods for measurement of EL in human serum samples have been developed [9]. Later, a time-resolved fluoroimmunoassay (TR-FIA) methodology for EL measurement was developed [10], and this method has been used in majority of the large, population-based studies investigating the relationship between human serum or plasma EL concentration and the risk of a particular disease [11–13]. The high sensitivity, wide working range, and easy to perform technique of the immunoassay have enabled rapid analyses of EL in large sample sets [10]. However, the suitability of the method has not yet been evaluated for samples expected to contain high EL concentrations (like after flaxseed consumption) or different ratio of EL enantiomers.

 In this pilot study, we measured for the first time the concentrations of EL enantiomers in serum from adult volunteers consuming their habitual diet and after flaxseed ingestion and evaluated whether the serum EL enantiomer profile was associated with the serum enterolignan concentrations. Moreover, the serum EL concentration levels obtained with TR-FIA and validated chromatographic methods were compared (GC-MS, HPLC-MS/MS, HPLC-coulometric electrode array detection (CEAD)) to evaluate the suitability of the immunoassay method for the analysis of serum samples after flaxseed ingestion.

## 2. Materials and Methods

### 2.1. Serum Samples

The used study protocol was reviewed and approved by the ethical committee of the Hospital District of Southwest Finland and all participants gave their informed consents. Blood samples were collected from five female and five male Finnish healthy volunteers, aged 22–58 years consuming their habitual diet (samples 1–5, and 6–10, resp.) and after eight days supplementation with 25 grams of crushed flaxseed per day (samples 11–15, and 16–20, resp.). The samples were collected 12 hours after the last dose of flaxseed. The serum was separated by centrifugation and stored at −20°C.

### 2.2. Analysis of Serum Enterolactone

The human serum samples were analyzed for EL concentrations in three laboratories (I-III) in Finland. The EL used for the standards in laboratories I and II was purchased from VTT Technical Research Centre of Finland (Espoo, Finland). In laboratory III, the EL used for standards was synthesized in Prof. Kristiina Wähälä's laboratory in the Department of Organic Chemistry at the University of Helsinki, Finland. 

In laboratory I, the enantiomers of EL in serum were determined with an HPLC-MS/MS method as previously described [7]. The serum ED and EL concentrations were determined using an HPLC-MS/MS method as previously described [14]. In laboratory II, the serum samples were analyzed with HPLC-CEAD [15] with slight modifications. Samples were purified prior to HPLC analysis with QAE-Ac^−^-ion exchange gel as described earlier for urine analysis [16]. Chromatographic separation was an isocratic 35-min run with the same eluents as published earlier [15], but the mobile phase consisted of 70% eluent B, and the quantification potential was 680 mV. Quantification was carried out with the standard addition method using blank plasma treated like a sample and containing added EL from 6.8 to 170 nM. Original estradiol glucuronide recovery standard was no longer needed because the standard addition method takes recovery corrections into account during the quantification. Samples which exceeded the calibration range were diluted with blank plasma prior to sample pretreatment to work on calibration range. In laboratory III, the serum samples were analyzed using GC-MS [8] but carrying out the hydrolysis subsequently without separate extraction of the sulfates and glucuronides, and with HPLC-CEAD [17], and TR-FIA [10] with further modifications [18]. Each sample was analyzed once with the previously validated chromatographic methods [8, 14–17]. In TR-FIA method, two parallels of each sample were analyzed [10, 18]. Due to limited volume of some of the serum samples, they were not available for all analyses.

### 2.3. Statistical Analyses

The statistical analyses were performed with Statistica software for Windows (Stat Soft, Tulsa, OK). The differences in serum (+) and (−)EL concentrations were analyzed with one-way analysis of variance followed by LSD post-hoc test. Correlation of the measured serum lignans was determined by Pearson correlation coefficient. The acceptable level of significance was set at *P* ≤ .05 for all analyses.

## 3. Results

In all analyzed human serum samples, the proportions of EL enantiomers varied interindividually. In all samples collected prior to the flaxseed ingestion, (−)EL dominated (Table 1). As expected, 8-day consumption of flaxseed increased significantly both serum enterodiol (ED) and EL concentrations of all individuals (*P* < .05). In half of the volunteers (2 women and 3 men), ED was the major serum enterolignan. Flaxseed ingestion increased the serum (+)EL concentrations significantly to 23–188-fold, resulting into its dominance in all samples. Also, a smaller (2–13-fold) increase in serum (−)EL concentration was observed after flaxseed consumption (*P* < .05). In samples collected after flaxseed consumption, (−)EL concentration correlated positively with serum (+)EL concentrations (*R* = 0.87, *P* < .05). However, the ED concentration did not correlate with the total EL or EL enantiomer concentrations. 

 The serum EL concentrations obtained with all the methods correlated with each other (*R* = 0.92–0.99, *P* < .05). In serum samples with relatively low EL concentrations (i.e., collected from subjects on their habitual diets), the average variation of the results obtained with chromatographic and TR-FIA methods was larger (CV% 35.2 and 42.0, resp.) than in samples with high EL concentrations collected after the flaxseed ingestion (CV% 14.4 and 33.4, resp.). After flaxseed ingestion, the lowest concentrations were measured with TR-FIA in all human serum samples (Table 2). Those were significantly (*P* < .05) lower than the concentrations obtained with chromatographic methods (Table 2). The highest concentrations were measured with HPLC-MS-MS (Table 2) but those were not statistically different from the concentrations measured with GC or HPLC-CEAD.

## 4. Discussion

In this pilot study, we demonstrate for the first time that the ratio of (+) and (−)EL in human serum varies significantly between individuals having their habitual diet. However, in all serum samples collected during habitual diet consumption, (−)EL predominated. This indicates that the majority of the dietary precursor plant lignans have the same configuration at the asymmetric carbons of the lactone ring as (−)EL. Flaxseed, however, contains mostly (+)secoisolariciresinol in diglycosylated form [19]. We have previously shown in rats that secoisolariciresinol diglycoside isolated from flaxseed is a precursor for (+)EL [7]. In this study, we demonstrate for the first time that (+)EL dominates also in human serum after the flaxseed ingestion. 

A small increase in serum (−)EL concentration was observed after the flaxseed consumption. This is likely due to (−)secoisolaricireresinol, a minor lignan in flaxseed [19], that is a parent compound for (−)EL formation in vivo [7]. Moreover, flaxseed contains (+)pinoresinol [19] which is further converted to (−)secoisolariciresinol [20, 21]. After flaxseed ingestion, the serum (−)EL concentrations correlated significantly with the (+)EL concentrations, despite the small individual differences in (−)EL serum concentrations. This suggests that the stereochemical structure of the plant lignan precursors in flaxseed is not the major determinant for the EL production capacity of the intestinal microbiota in human subjects.

In serum samples with high EL concentrations (i.e., after the flaxseed ingestion), the measured EL concentrations were significantly lower with the immunoassay than with the chromatographic methods. The dominance of (+)EL in samples collected after the flaxseed consumption may have resulted in underestimation of EL concentrations measured with the immunoassay method, as the antibodies are often stereoselective [22]. Preliminary results suggest that at high concentrations the antibody reacts predominantly with (−)EL. Thus, for samples expected to contain high EL concentrations with variable enterolactone enantiomer ratios, other analytical methods should be preferred.

## 5. Conclusions

We show here for the first time that the ratio of human serum (+) and (−)EL enantiomers varies and can be significantly altered by dietary supplementation with flaxseed. Moreover, we showed that the immunoassay is not an optimal quantitation method for samples with very high EL concentrations or with significantly altered ratio of EL enantiomers like the ones collected after the flaxseed ingestion. Therefore, chromatographic quantitation methods should be preferred in flaxseed intervention studies.

## Figures and Tables

**Table 1:** Concentrations of enterolactone (EL) enantiomers and enterodiol (ED) in human serum samples collected before and after flaxseed supplementation.

	(+)EL	(−)EL	ED
Sample^#^		nmol/L	
1W	22.3	32.7	nq
2W	6.7	15.8	6.1
3W	19	57.4	2.6
4W	4.9	11.9	nq
5W	5.3	5.7	nq
6M	6.3	10.9	nq
7M	41.3	44.5	nq
8M	10.6	11.9	nq
9M	9.2	13.1	nq
10M	4.3	5.7	nq

11W	1332.9	101.9	649.5
12W	977.7	81.6	1022.1
13W	1645.1	205.4	372.3
14W	239.9	22.3	369.6
15W	226.6	37.2	645.4
16M	116.6	44	968.9
17M	1063.5	82.5	237.2
18M	580.2	40.3	77.7
19M	467.9	58.4	1143.9
20M	140.5	81.1	1828.8

^#^Samples 1–10 are collected from healthy Finnish women (W) and men (M) having their habitual diet and samples, 11–20 are from the same individuals after 8 days of flaxseed supplementation (25 grams per day). Samples with ED levels below the limit of quantification are marked nq (non quantifiable).

**Table 2:** Human serum EL concentrations (nM) before and after flaxseed ingestion measured by chromatographic and TRFIA methods.

Laboratory	I	II	III		
Method	HPLC-MS/MS	HPLC-CEAD	GC-MS	HPLC-CEAD	TR-FIA
Samples^#^					
1W	55	33.9	NA	32.7	28.1
2 W	22.5	12.3	NA	14.9	12.1
3 W	76.4	40	NA	53.1	28.4
4 W	16.8	11.9	NA	13.9	11.2
5 W	11	6.7	NA	12.1	9
6 M	17.2	6.9	NA	12.6	5.5
7 M	85.8	52.4	NA	54	52.9
8 M	22.5	14.5	NA	15.1	14.3
9 M	22.3	9.2	NA	13.1	10.2
10 M	10	7.3	NA	29.3	8.2

11 W	1434.8	NA	NA	NA	NA
12 W	1059.3	930.9	886.2	1016.7	359.6
13W	1850.5	NA	NA	NA	NA
14W	262.2	225.7	260.3	235.7	105.2
15W	263.8	220.8	190.8	219.8	127.9
16M	160.6	110	144.4	128.3	54.4
17M	1146	NA	NA	NA	NA
18M	620.5	NA	NA	NA	NA
19M	526.3	438	376.2	421.8	201.2
20M	221.6	161.7	167.6	142.6	108.4

^#^Samples 1–10 are collected from healthy Finnish volunteers having their habitual diet and samples 11–20, are from the respective individuals after 8 days of flaxseed supplementation (25 grams per day). Samples not available for analysis due to a limited sample volume are marked as NA. Samples collected from women are marked with W and those collected from men with M.
